# Modulating empathy with tDCS: dissociable roles of rTPJ and lDLPFC

**DOI:** 10.1017/S0033291726104218

**Published:** 2026-04-28

**Authors:** Xiaodong Li, Zekun Guo, Jialin Ye, Xilin Yang, Xuejing Lu, Weiwei Peng

**Affiliations:** 1 School of Psychology, Shenzhen University, Shenzhen, China; 2 CAS Key Laboratory of Mental Health, Institute of Psychology, Chinese Academy of Sciences, Beijing, China; 3 Department of Psychology, University of Chinese Academy of Sciences, Beijing, China

**Keywords:** empathy, left dorsolateral prefrontal cortex, multimodal decoding, right temporoparietal junction, transcranial direct current stimulation

## Abstract

**Background:**

Empathy relies on distinct but interacting processes for representing others’ states and regulating self-oriented affect. Neuroimaging studies implicate the right temporoparietal junction (rTPJ) in perspective-taking and the left dorsolateral prefrontal cortex (lDLPFC) in emotion regulation, yet causal evidence from neuromodulation remains limited. This study compared the effects of rTPJ- and lDLPFC-targeted transcranial direct current stimulation (tDCS) on empathy across multiple contexts and modalities.

**Methods:**

In Study 1, participants performed a static pain empathy task following anodal or sham tDCS over the rTPJ or lDLPFC, with electroencephalography recorded. In Study 2, participants viewed autobiographical videos depicting positive, negative, and neutral events before and after stimulation, while heart rate variability (HRV) was assessed. Machine learning-based decoding integrated behavioral and physiological data to evaluate the ‘readability’ of empathic states.

**Results:**

rTPJ-tDCS enhanced cognitive empathy across tasks, increasing empathic ratings and late positive potential amplitudes in the pain empathy task, and enhancing the subjective sense of content and emotion understanding in the video task. lDLPFC-tDCS selectively increased HRV in the video task, consistent with greater autonomic flexibility, without altering explicit ratings. Decoding analyses converged with these findings: rTPJ stimulation increased classification accuracy of targets’ emotional states, indicating stronger alignment between empathic responses and others’ emotional cues, whereas lDLPFC stimulation reduced accuracy, suggesting regulation-related attenuation of overt emotional signals.

**Conclusions:**

These findings provide causal evidence for rTPJ supporting cross-context cognitive empathy and lDLPFC modulating autonomic regulation. Multi-context, multimodal assessment delineated distinct target-specific profiles, informing precision neuromodulation strategies for empathy-related deficits and regulation needs.

## Introduction

Empathy, the capacity to resonate with and respond to others’ experiences, is a cornerstone of human social interaction (Singer & Lamm, 2009). Rather than a unitary construct, empathy entails two interrelated processes (Davis, 1983; Decety & Lamm, 2006; Zaki & Ochsner, 2012). One is other-oriented, encompassing perspective-taking, understanding others’ emotions, and concern for their well-being. The other is self-oriented, involving the regulation of personal distress and the downregulation of aversive reactions. These complementary processes jointly promote prosocial behavior, effective communication, and social bonding (Batson, Early, & Salvarani, 1997; Eisenberg & Fabes, 1990): the former fosters social connection, while the latter maintains emotional balance to prevent empathic engagement from becoming overwhelming. Dysregulation of either process can be detrimental. Deficits in other-oriented empathy are characteristic of conditions such as autism spectrum disorder (Baron-Cohen & Wheelwright, 2004), schizophrenia (Bragado-Jimenez & Taylor, 2012), and psychopathy (Blair, 2013), whereas excessive self-oriented distress contributes to burnout and secondary traumatization (Figley & Ludick, 2017). These observations highlight the importance of modulating empathy’s dual facets, enhancing other-oriented understanding when social connection is critical, and strengthening self-oriented regulation when resilience is needed (Wu et al., 2024).

Non-invasive brain stimulation (NIBS) offers a promising approach to modulate empathy by directly targeting neural circuits supporting its self- and other-oriented components (Hetu, Taschereau-Dumouchel, & Jackson, 2012). Among NIBS techniques, transcranial direct current stimulation (tDCS) is particularly attractive due to its safety, cost-effectiveness, and ease of application in both research and clinical settings (Nitsche & Paulus, 2000; Woods et al., 2016). Two cortical regions have emerged as critical and complementary candidates: the right temporoparietal junction (rTPJ) and the left dorsolateral prefrontal cortex (lDLPFC). The rTPJ is a core node of the social-cognition network (Decety & Lamm, 2007; Saxe & Kanwisher, 2003), supporting perspective-taking, self-other distinction, and mental-state attribution, and thus underpinning other-oriented empathic representation. In contrast, the lDLPFC is central to emotion-regulation networks (Dorfel et al., 2014; Zhao et al., 2021), exerting top-down control to regulate self-focused distress and support sustained, goal-directed empathic engagement. Accordingly, these regions map onto distinct facets of empathy: rTPJ primarily supports representing others’ states (Kubit & Jack, 2013), whereas lDLPFC primarily supports regulation of one’s own affective responses (Travassos et al., 2020). Although prior tDCS studies suggest that stimulation of these regions can influence perspective-taking or distress regulation, findings on empathy remain inconsistent and highly context-dependent (Bahji, Forth, Yang, & Khalifa, 2021; Coll, Tremblay, & Jackson, 2017; Mai et al., 2016; Rego et al., 2015; Snowdon & Cathcart, 2018; Wang, Wang, Hu, & Li, 2014). This variability raises a key question: whether rTPJ and lDLPFC stimulation reliably modulates empathy across contexts, and whether they exert dissociable effects on other- versus self-oriented processes.

Despite growing interest in targeting the rTPJ and lDLPFC with tDCS, existing studies have relied predominantly on behavioral measurements. While informative, such indices are susceptible to social desirability and limited introspective access (Pang et al., 2023; Sunahara et al., 2022) and fail to capture implicit neural and autonomic processes involved in empathic engagement. Although no single physiological marker is specific to empathy, different indices provide process-sensitive access to complementary components of empathic processing. Specifically, late positive potentials (LPPs) from electroencephalography (EEG) index sustained attentional allocation and evaluative processing of emotionally and socially salient stimuli (Hajcak, MacNamara, & Olvet, 2010; Schupp, Flaisch, Stockburger, & Junghofer, 2006) and have been consistently linked to processing others’ affective states during pain observation and social–emotional evaluation (Li et al., 2020, 2025; Peng et al., 2019; Toppi et al., 2022). In contrast, heart rate variability (HRV) derived from electrocardiography (ECG) reflects autonomic flexibility and emotion regulation capacity (Appelhans & Luecken, 2006; Laborde, Mosley, & Thayer, 2017; Thayer & Lane, 2009), capturing self-oriented regulatory processes that may constrain or support empathic engagement (Di Bello et al., 2020; Williams et al., 2015). Together with behavioral measures, LPP and HRV allow dissociation between representational and regulatory components of empathy across contexts. Moreover, few studies have adopted computational approaches beyond univariate analyses. Decoding methods that integrate behavioral and EEG/ECG signals provide a multivariate index of the ‘readability’ of empathic states, offering complementary insight into how neuromodulation shapes the coherence and external interpretability of empathic processing (Nikolova, Petkova, Manolova, & Georgieva, 2018; Woo, Chang, Lindquist, & Wager, 2017; Zhang, Yin, Chen, & Nichele, 2020).

**Figure 1.**
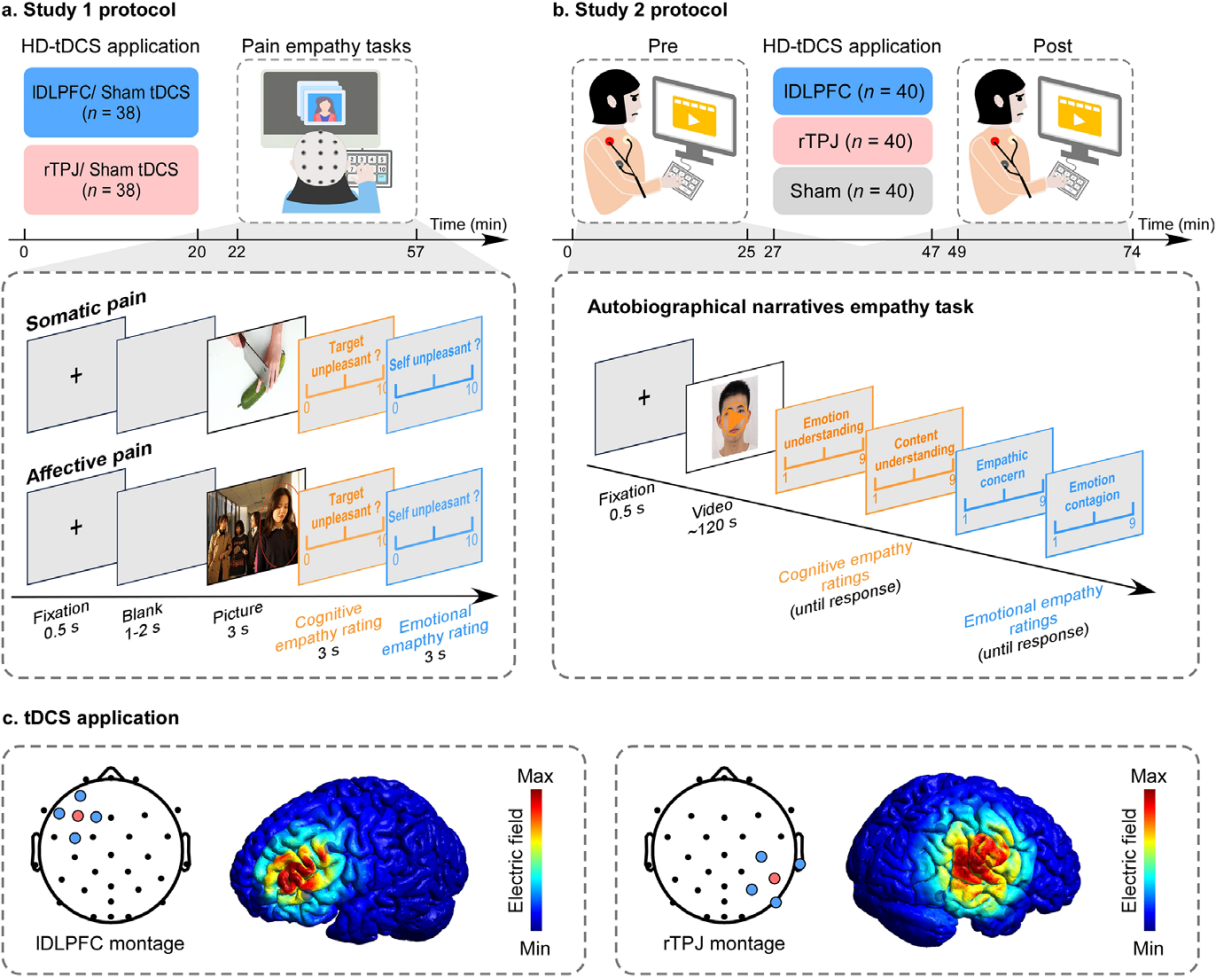
Overview of experimental design and stimulation protocol. (a) *Study 1 protocol.* Participants received both real and sham tDCS over either the left dorsolateral prefrontal cortex (lDLPFC; blue) or the right temporoparietal junction (rTPJ; red), in two sessions separated by 7 days. Stimulation was delivered at 1.5 mA for 20 min (real) or 1 min (sham), with order counterbalanced. Following stimulation, participants performed two picture-based empathy tasks (somatic pain, affective pain; order counterbalanced) while an EEG was recorded. Each trial consisted of a fixation cross (0.5 s), a blank screen (1–2 s), and a painful (limb injury) or nonpainful/social exclusion picture (3 s), followed by ratings of the protagonist’s pain intensity and participants’ own unpleasantness (0–10 scale). (b) *Study 2 protocol.* Participants received a single session of anodal tDCS over the lDLPFC (blue), rTPJ (red), or sham (gray). Before (pre) and after (post) stimulation, they completed an autobiographical narratives empathy task with concurrent ECG recording. Each trial included a fixation cross (0.5 s), a 2-min video of a protagonist describing a positive, negative, or neutral autobiographical event, and subsequent ratings of content understanding, emotional understanding, empathic concern, and emotional contagion (0–10 scale). (c) *Stimulation montage and electric field simulation.* The montage used one central anodal electrode (red) over the target site (lDLPFC or rTPJ) and four return electrodes (blue) arranged radially. Finite-element modeling showed the electric field distribution, with color gradients indicating field strength from minimum (blue) to maximum intensity (red).

To address these gaps, we examined the effects of rTPJ- and lDLPFC-tDCS on empathic processing using multi-context paradigms and multimodal outcome measures, providing causal evidence for their roles in empathy. Two complementary empathy paradigms were employed to capture distinct components of empathic processing across temporal and contextual scales. The picture-based pain empathy task probed rapid, stimulus-driven responses to others’ somatic and affective pain cues and was paired with EEG recordings to characterize attentional and affective neural processes. In contrast, the autobiographical narrative task involved sustained engagement with ecologically valid emotional experiences, placing greater demands on perspective-taking and integrative emotion understanding, and was therefore paired with ECG recordings to index autonomic regulation during prolonged empathic engagement. Across two experiments, participants received anodal tDCS and then completed either the picture-based task (Study 1; Figure 1a) or the narrative-based task (Study 2; Figure 1b). Behavioral, EEG/ECG, and computational decoding outcomes were assessed. This dual-paradigm design allowed us to test whether neuromodulatory effects generalized across empathic contexts rather than reflecting task-specific modulation.

## Methods

### Study 1

#### Participants

Eighty healthy adults (43 females; mean age = 20.86 years) participated in Study 1. All participants were right-handed, had normal or corrected-to-normal vision, and reported no history of acute or chronic pain, neurological disorders, cerebrovascular disease, or psychiatric conditions. They were randomly assigned to receive tDCS targeting either the lDLPFC or the rTPJ, with 40 participants in each group. Four individuals were excluded due to missing data (*n* = 2) or equipment failure (*n* = 2), resulting in a final sample of 76 participants (38 per group). Demographic characteristics are presented in Table 1. All participants provided written informed consent in accordance with the Declaration of Helsinki, and the study protocol was approved by the Ethics Committee of the Medical School of Shenzhen University.

**Table 1.** Demographic and psychometric characteristics of participants in Study 1

	lDLPFC group	rTPJ group	Statistics *t_74_*/*χ*^2^*_(1)_*
Sample size (*n*)	38	38	
Gender (F/M)	21/17	21/17	< 0.001
Age (years)	20.76 ± 0.31	20.97 ± 0.31	−0.48
Trait empathy (IRI)			
Perspective taking	13.42 ± 0.40	13.16 ± 0.48	0.48
Fantasy	15.61 ± 0.47	16.11 ± 0.48	−0.74
Empathic concerns	15.90 ± 0.47	15.90 ± 0.46	<0.001
Personal distress	10.74 ± 0.54	10.18 ± 0.54	0.72
Trait anxiety (STAI-T)	32.47 ± 1.47	29.92 ± 1.54	1.20

*Note*: Values are presented as Mean ± SEM. Group comparisons were conducted using Chi-square tests or independent-sample *t*-tests. F = female; M = male. Trait empathy was assessed with the Interpersonal Reactivity Index (IRI), and trait anxiety was assessed with the Trait subscale of the State–Trait Anxiety Inventory (STAI-T).

#### General experimental procedure

Study 1 employed a 2 (tDCS target: lDLPFC versus rTPJ; between-subjects) × 2 (tDCS type: real versus sham; within-subjects) × 2 (stimulation type: painful versus nonpainful; within-subjects) × 2 (task type: somatic versus affective; within-subjects) factorial design. For each group of participants (lDLPFC or rTPJ), they completed both real and sham stimulation sessions in a counterbalanced crossover design, with a 7-day washout period between sessions. As illustrated in Figure 1a, each tDCS session lasted 20 min. Immediately following each stimulation session, participants performed two pain empathy tasks (somatic pain and affective pain empathy) while EEG activity was continuously recorded. In each task, participants viewed images depicting somatic or affective pain or nonpain situations, and rated their level of empathy. Before the experimental sessions, participants completed questionnaires assessing trait empathy and anxiety, including the Interpersonal Reactivity Index (IRI) and the trait subscale of the State–Trait Anxiety Inventory (STAI-T). As shown in Table 1, the lDLPFC and rTPJ groups were well matched in terms of their psychological characteristics.

#### tDCS administration

A 4 × 1 Multichannel Stimulation Adaptor (Model 4 × 1-C3A; Soterix Medical In., New York, NY) was employed on the scalp. Electrode placement followed the international 10–20 EEG system, with 5 Ag-AgCl sintered ring electrodes arranged in a 4 × 1 montage over the target site. The central electrode was placed at F3 with the four surrounding return electrodes at AF3, F1, F5, and FC3 for the lDLPFC stimulation (Nikolin et al., 2015), and placed at CP6, surrounded by C4, T8, P4, and P8 for the rTPJ stimulation (Qiao et al., 2020). The center-to-center distance was 3–5 cm, and skin-electrode impedance values were verified to be <1.0 quality unit. For real stimulation, a 1.5 mA direct current was delivered for 20 min. Sham stimulation mimicked real tDCS for 1 min at the beginning and 1 min at the end of the session, while no current was applied for the remaining.

To verify the stimulation protocol, finite-element modeling of the electric field based on a standard head model using the Montreal Neurological Institute (MNI) 152 template was performed in ROAST v3.0 (https://github.com/andypotatohy/roast) using the electrode montage applied in both studies. The simulations indicated that the induced electric field was primarily concentrated beneath the central electrode, with maximal intensity localized at the lDLPFC ([−45, 31, 30], Brodmann area 9, peak E-field = 0.15 V/m) and rTPJ ([62, −34, 32], Brodmann area 40, peak E-field = 0.36 V/m) targets, respectively. As shown in Figure 1c, the field strength gradually declined with increasing distance from the center, confirming that this montage effectively focused current delivery to the intended cortical regions.

#### Pain empathy task

Study 1 employed two established paradigms to assess empathy for somatic and affective pain. For the somatic pain empathy task, 60 color images of limbs in painful (*n* = 30) or nonpainful (*n* = 30) everyday situations were used (Meng et al., 2013). Painful and nonpainful images depicted matched scenarios, with luminance, contrast, and color controlled across pairs. For the affective pain empathy task, 60 color images depicted either social exclusion (*n* = 30) or neutral (*n* = 30) situations (Zheng et al., 2022). Social exclusion images showed a protagonist with sad or upset expressions while other individuals (3–4) interacted or laughed together; neutral images showed groups of two to five individuals with neutral expressions and no social interaction. Visual properties were matched across pairs.

Stimuli were presented using E-Prime 3.0 (Psychology Software Tools, Pittsburgh, PA). Each trial began with a 500 ms fixation, followed by a blank screen (1000–2000 ms), and then a 3000 ms image (Figure 1a). Participants were asked to provide two ratings on a 0–10 numeric scale (0 = no unpleasantness, 10 = extreme unpleasantness): (1) ‘How much unpleasantness is the person in the picture in?’ and (2) ‘How much unpleasantness do you experience while viewing the picture?’ The first question targeted participants’ cognitive evaluation of the protagonist’s pain, whereas the second assessed affective sharing. The inter-trial interval varied randomly between 800 and 1200 ms. Each task consisted of 60 trials and lasted approximately 12 minutes.

#### EEG recording

Continuous EEG signals were continuously recorded during the pain empathy task using 32 Ag–AgCl electrodes arranged according to the international 10–20 system (ANT Neuro, The Netherlands; band-pass filter: 0.01–100 Hz; sampling rate: 1000 Hz). CPz served as the online reference, and FCz as the ground electrode. Electrode impedances were maintained below 10 kΩ.

#### EEG data analysis

EEG data were preprocessed using EEGLAB v21.0 (Delorme & Makeig, 2004). Continuous data were band-pass filtered between 0.1 and 30 Hz. For each task, epochs time-locked to picture onset were extracted from −1000 to 3000 ms, and baseline-corrected using the −1000 to 0 ms prestimulus interval. EEG epochs were visually inspected, and epochs containing transient jumps or channel-specific artifacts were manually excluded. On average, 2.00 ± 1.37 epochs were rejected per participant, accounting for less than 3% of the total EEG epochs. Subsequently, artifacts due to eye blinks or movements were corrected using independent component analysis (ICA). Following artifact correction, a second baseline adjustment was applied, and data were re-referenced to the common average reference.

For each participant, epochs belonging to the same condition (8 in total: 2 tDCS type [real versus sham] × 2 task types [somatic versus affective] × 2 stimulation types [pain versus nonpain]) were averaged, yielding eight ERP waveforms time-locked to picture onset. Group-level waveforms and scalp topographies were generated by spline interpolation. Analyses focused on the LPP, a component closely associated with pain empathy (Coll, 2018; Li et al., 2020; Peng et al., 2019). LPP amplitude was quantified as the mean voltage at the centroparietal region (CP1 and CP2) within two time windows: 500–1000 ms (early LPP) and 1000–3000 ms (late LPP) post-stimulus onset.

#### Statistical analysis

All statistical analyses were conducted using SPSS 22.0 (IBM Corp., Armonk, NY). Demographic and psychological measures (trait empathy and anxiety) were compared between the lDLPFC and rTPJ groups using χ^2^ tests or independent-samples *t*-tests.

Behavioral empathic responsiveness was quantified using the area under the curve (AUC), a nonparametric index from signal detection theory (SDT). Following a rating-based SDT approach (Clark & Yang, 1974), unpleasantness ratings (0–10 NRS) for painful and nonpainful stimuli were treated as implicit decision criteria (Macmillan, 2002). For each possible criterion (e.g. ≥5), trials meeting the criterion in the painful condition were counted as hits, and those in the nonpainful condition as false alarms. Hit and false alarm rates were calculated for each criterion, and the AUC was obtained by plotting hit rates against false alarm rates across all criteria. Higher AUC values indicate better discrimination between painful and nonpainful stimuli, independent of individual response bias (Hautus, Macmillan, & Creelman, 2021). Similarly, neural empathic responsiveness was defined as the difference in LPP amplitudes between painful and nonpainful conditions (painful−nonpainful). Both behavioral and neural indices were submitted to repeated-measures analysis of variance (ANOVA), with tDCS target (lDLPFC versus rTPJ) as a between-subject factor, and tDCS type (real versus sham), task type (somatic versus affective), and stimulation type (painful versus nonpainful) as within-subject factors.

#### Decoding painful versus nonpainful events

To test whether tDCS modulated the discriminability of empathic responses, machine learning classifiers were trained to distinguish painful from nonpainful stimuli using both behavioral ratings and EEG features. EEG features were extracted a priori from centroparietal electrodes and consisted of early and late LPP amplitudes, given their well-established sensitivity to the processing of others’ affective states (Coll, 2018; Li et al., 2020; Liu et al., 2012; Peng et al., 2019). All features were entered into the classifiers on their original scale, without standardization or normalization. Five frequently-used algorithms were implemented (Hemakom, Atiwiwat, & Israsena, 2023; Talukdar, Gogoi, & Singh, 2023): Logistic Regression (LR), k-Nearest Neighbors (KNN), Random Forest (RF), Support Vector Machine (SVM), and Naïve Bayes (NB). Leave-one-out cross-validation (LOOCV) was applied to maximize robustness given the sample size. Classification performance was evaluated using overall accuracy, receiver operating characteristic (ROC) curves, and Cohen’s Kappa, providing complementary indices of discriminability.

### Study 2

#### Participant

Study 2 included 120 healthy adults (65 females; mean age = 20.03 years), meeting the same inclusion criteria as in Study 1. Participants were randomly assigned to the lDLPFC, rTPJ, or sham stimulation group (n = 40 each). Demographic characteristics, summarized in Table 2, were well matched across groups. All participants provided written informed consent, and the protocol was approved by the Ethics Committee of the Medical School of Shenzhen University.

**Table 2.** Demographic and psychometric characteristics of participants in Study 2

	lDLPFC group	rTPJ group	Sham group	Statistics *F_2, 117_*/*χ*^2^*_(2)_*
Sample size (*n*)	40	40	40	
Gender (F/M)	21/19	21/19	23/17	0.27
Age (years)	19.70 ± 0.30	20.35 ± 0.31	20.05 ± 0.31	1.11
Trait empathy (QCAE)				
Cognitive empathy	56.93 ± 1.14	56.98 ± 0.95	55.43 ± 1.01	0.72
Affective empathy	32.38 ± 0.94	31.48 ± 0.74	32.48 ± 0.75	0.46
Trait anxiety (STAI-T)	41.15 ± 1.31	41.95 ± 1.72	41.33 ± 1.72	0.07

*Note*: Data are expressed as Mean ± SEM. Statistical analyses were performed using Chi-square tests or one-way ANOVA. F = female; M = male; Trait empathy was assessed with the Questionnaire of Cognitive and Affective Empathy (QCAE); Trait anxiety was assessed with the Trait subscale of the State–Trait Anxiety Inventory (STAI-T).

#### Experimental procedure

Study 2 employed a 3 (tDCS type: lDLPFC versus rTPJ versus sham; between-subjects) × 3 (stimulus type: positive versus negative versus neutral; within-subjects) × 2 (time: pre- versus post-stimulation; within-subjects) factorial design. The study design is illustrated in Figure 1b. Participants were randomly assigned to one of three parallel groups (lDLPFC, rTPJ, or sham tDCS) and completed an autobiographical narratives empathy task both before and after the assigned stimulation. ECG activity was recorded continuously during the task. In the empathy task, participants viewed a series of ~2-min video clips in which protagonists recounted personal experiences with positive (e.g. participating in an international choir), negative (e.g. losing a close friend), or neutral (e.g. discussing study plans) emotional content. After each clip, participants rated their level of empathy toward the protagonist. Prior to the experimental session, all participants completed questionnaires assessing trait empathy and anxiety, including the Questionnaire of Cognitive and Affective Empathy (QCAE) and the trait subscale of the STAI. As shown in Table 2, the lDLPFC, rTPJ, and sham groups were well matched in terms of their psychological characteristics.

#### Autobiographical narratives empathy task

This task was designed to assess both cognitive and affective components of empathy in response to emotionally valenced autobiographical narratives. The stimuli consisted of 24 video clips in which protagonists recounted personal experiences with positive, negative, or neutral emotional content. Luminance, contrast, and color properties were matched across categories to minimize low-level visual differences.

We initially collected 84 autobiographical video clips and screened them through three trained psychologists using the following exclusion criteria: (1) ambiguous or mixed emotional valence; (2) unclear or difficult-to-follow narration; (3) withdrawal requests from the protagonist; and (4) narratives not based on the protagonist’s own experiences. This screening yielded 37 candidate clips. These clips were then evaluated by 10 independent raters on both technical quality (voice clarity, narrative coherence, nervousness, speech rate, and background noise) and emotional characteristics (valence and arousal). The same raters further assessed each clip on four empathy-related dimensions (content understanding, emotional understanding, empathic concern, and personal distress), using a 1–5 scale (1 = not at all, 5 = extremely). Based on these evaluations, 24 clips (15 female protagonists) were selected, which showed clear and significant differences in emotional valence across categories. Repeated-measures ANOVAs confirmed robust effects of stimulus type (*F₂,₁₈* = 19.66–55.11, *p* < 0.001, *η_p_^2^* = 0.69–0.86), with positive and negative clips eliciting greater content and emotional understanding than neutral clips, and negative clips inducing stronger empathic concern and personal distress than both positive and neutral clips.

In the main experiment, participants completed the task before and after tDCS application (Figure 1b), each containing 12 trials (4 per category). Each trial began with a 2000-ms fixation cross, followed by a 2-min video clip from one of the three emotional categories. After viewing each clip, participants provided four ratings on a 1–9 numeric scale (1 = not at all, 9 = extremely): (1) ‘How well do you understand what the protagonist described?’ (Content understanding); (2) ‘How accurately do you perceive the protagonist’s emotions?’ (Emotion understanding); (3) ‘How much concern do you feel for the protagonist?’ (Empathic concern); and (4) ‘To what extent do the protagonist’s emotions influence your own feelings?’ (Emotional contagion). The first two questions indexed cognitive empathy, while the latter two reflected affective empathy. The total task duration was approximately 27 minutes.

#### ECG recording and data analysis

Continuous ECG signals were continuously recorded throughout the autobiographical narratives empathy task. Three disposable Ag/AgCl electrodes were placed in a modified Lead II configuration (right and left mid-clavicle, and the left lower rib). Signals were acquired using an ECG100C amplifier connected to a BIOPAC MP150 system (BIOPAC Systems Inc., Goleta, CA, USA) at a sampling rate of 1000 Hz, ensuring accurate capture of QRS complex components (Allen, Chambers, & Towers, 2007).

Raw ECG signals were denoised using Ensemble Empirical Mode Decomposition (Wu & Huang, 2009), a data-driven method suitable for nonlinear and non-stationary physiological signals (Han, Lin, & Xu, 2017). R-peaks were identified using an extreme point detection algorithm, followed by manual correction to ensure accuracy. The resulting RR interval (RRI) series was used for HRV analysis across time, frequency, and nonlinear domains. In the time domain, we calculated HR (averaged heart rate), SDNN (overall variability reflecting sympathetic and parasympathetic inputs), RMSSD (short-term beat-to-beat variability reflecting vagal activity), and pNN50 (the percentage of NN50 quantifying short-term vagal modulation) (Rocha et al., 2024). Frequency-domain measures were derived using Fast Fourier Transform, including low-frequency (LF: 0.04–0.15 Hz; baroreflex-related), high-frequency (HF: 0.15–0.40 Hz; parasympathetic activity), and the LF/HF ratio (sympathovagal balance) (Shaffer & Ginsberg, 2017). Nonlinear indices included sample entropy (signal complexity) and Poincaré plot parameters (SD1, SD2, SD1/SD2 ratio) to capture autonomic dynamics across short- and long-term scales (Gao et al., 2022). These indices were computed for each participant and condition to characterize autonomic responses during empathy-related processing.

#### Statistical analysis

All statistical analyses were conducted using SPSS 22.0 (IBM Corp., Armonk, NY). Demographic and psychological measures (trait empathy and anxiety) were compared across the lDLPFC, rTPJ, and sham groups using χ^2^ tests for categorical variables and one-way ANOVA for continuous variables.

For the main analyses, single-trial empathic ratings and HRV measures corresponding to the same experimental condition were averaged within each participant, yielding six condition-specific values (3 stimulation types × 2 time points). Behavioral outcomes included content recognition, emotion recognition, empathic concern, and emotion contagion, while physiological outcomes included HRV indices. Change scores (post–pre) were computed for each participant and condition. These change scores were submitted to a two-way mixed-design ANOVA with a between-subjects factor of tDCS type (lDLPFC versus rTPJ versus sham) and a within-subjects factor of stimulus type (positive versus negative versus neutral). Significant main effects or interactions were followed by Bonferroni-corrected post hoc tests to control for multiple comparisons.

#### Decoding autobiographical event valence

To evaluate whether tDCS modulated empathic processing of autobiographical narratives, we trained machine learning classifiers to discriminate event valence (positive, neutral, negative) using ECG features and behavioral ratings. Consistent with Study 1, five algorithms were tested (including LR, RF, SVM, KNN, and NB), with leave-one-out cross-validation applied for training and testing (Saboor et al., 2022). Classification performance was evaluated using overall accuracy, ROC curves, and Cohen’s Kappa as a complementary index.

## Results

### Study 1

**Figure 2.**
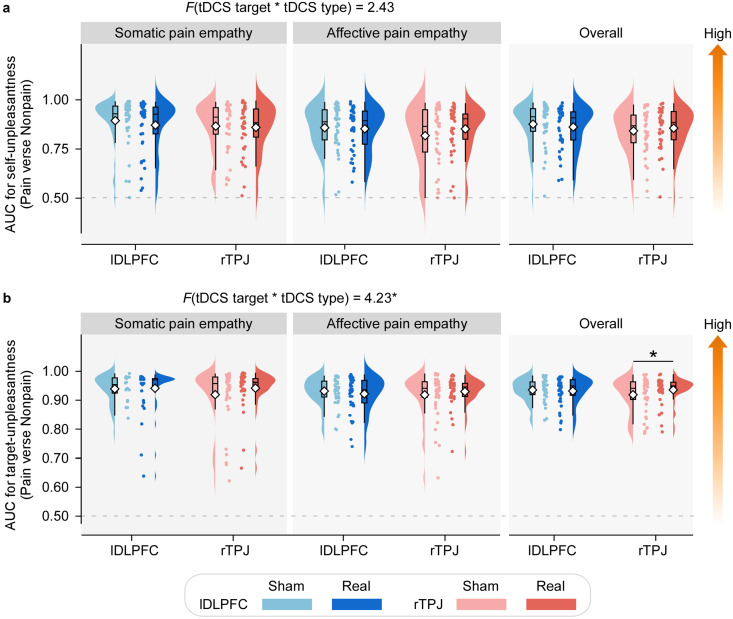
Effects of tDCS on pain empathic responsiveness. Raincloud plots show the distribution of area under the curve (AUC) values indexing pain empathic responsiveness – that is, the ability to discriminate painful from nonpainful conditions – for (a) self-unpleasantness ratings and (b) other-unpleasantness ratings. Data are presented separately for the somatic and affective pain empathy tasks, as well as for their combined average. Each raincloud plot integrates a violin plot (probability density), a box plot (interquartile range with median: black line), mean values (diamonds), and individual data points. Compared with sham stimulation, real rTPJ-tDCS significantly increased AUC values for other-unpleasantness ratings. *p < 0.05.

Figure 2 illustrates the effects of tDCS on behavioral empathic responsiveness. For self-unpleasantness AUC values (indexing emotional empathic responsiveness), a significant main effect of task type was observed (*F₁,₇₄* = 5.19, *p* = 0.026, *η_p_^2^* = 0.07; Figure 2a), with larger AUC values in the somatic pain empathy task than in the affective pain empathy task. Other main effects or interactions were nonsignificant. It suggests that emotional empathic responsiveness was generally greater in empathy for somatic pain than for affective pain. For other-unpleasantness AUC values (indexing cognitive empathic responsiveness), a significant interaction between tDCS target and tDCS type emerged (*F₁,₇₄* = 4.23, *p* = 0.043, *η_p_^2^* = 0.05; Figure 2b). Post hoc comparisons revealed that real rTPJ-tDCS elicited significantly greater AUC values more than sham stimulation (*p* = 0.020), whereas no significant difference was found between real and sham stimulation in the lDLPFC group (*p* = 0.604). These results indicate that rTPJ-tDCS selectively enhanced cognitive empathic responsiveness, independent of task type.

**Figure 3.**
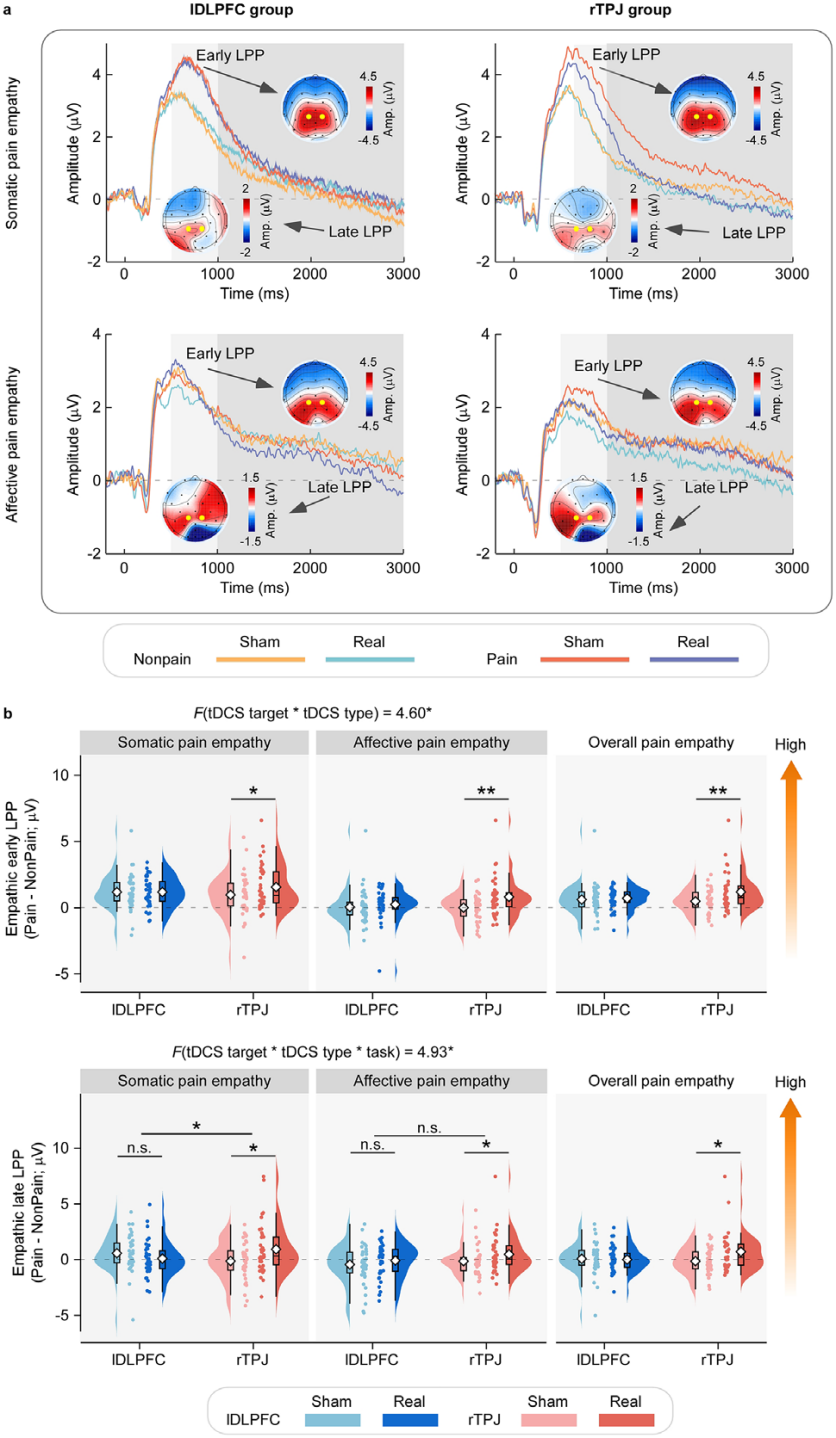
Effects of tDCS on neural responsiveness during pain empathy (a) *Grand-averaged ERP waveforms and scalp topographies.* Signals are shown for each tDCS target (lDLPFC, rTPJ) and task type (somatic versus affective pain empathy), elicited by painful and nonpainful pictorial stimuli following real or sham stimulation. Waveforms are plotted for centroparietal electrodes (CP1, CP2; highlighted with enlarged white dots). Early and late LPP windows are marked by light and dark gray shading, respectively. (b) *Effects of tDCS on empathic LPP amplitudes.* Raincloud plots show early and late LPP amplitudes (Pain – Nonpain) for each tDCS target (lDLPFC: blue; rTPJ: red) and tDCS type (real: darker color; sham: lighter color). Data are displayed separately for somatic and affective tasks, as well as for the combined average. Raincloud plots integrate violin plots (probability density), box plots (IQR, median: black line), mean values (diamonds), and individual data points. Compared with sham, real rTPJ-tDCS significantly increased both early and late empathic LPP amplitudes across tasks. *p < 0.05, **p < 0.01; n.s., *p* > 0.05.

Grand-average ERP waveforms and scalp topographies for early and late LPP amplitudes are shown in Figure 3a. Both painful and nonpainful stimuli evoked robust LPP responses with maximal activity over centroparietal sites. The ΔLPP amplitude (painful–nonpainful) results are summarized in Figure 3b. A significant interaction between tDCS target and tDCS type was observed for both early ΔLPP (*F₁,₇₄* = 4.60, *p* = 0.035, *η_p_^2^* = 0.06) and late ΔLPP (*F₁,₇₄* = 5.93, *p* = 0.017, *η_p_^2^* = 0.07). Post hoc comparisons revealed that real rTPJ-tDCS improved ΔLPP amplitudes than sham stimulation (early: *p* = 0.001; late: *p* = 0.002), whereas the lDLPFC group showed no such difference (early: *p* = 0.641; late: *p* = 0.799). These results suggest that rTPJ-tDCS, but not lDLPFC-tDCS, enhances both early and late ERP markers of empathic differentiation.

A significant three-way interaction of tDCS target × tDCS type × task type was found for the late ΔLPP amplitude (*F₁,₇₄* = 4.93, *p* = 0.029, *η_p_^2^* = 0.06). Post hoc analyses revealed that, in the somatic pain empathy task, a significant tDCS target × tDCS type interaction was present (*F₁,₇₄* = 9.27, *p* = 0.003, *η_p_^2^* = 0.11): real rTPJ-tDCS significantly increased late ΔLPP amplitudes compared with sham (*p* = 0.004), whereas no such difference occurred for lDLPFC-tDCS (*p* = 0.180). In contrast, in the affective pain empathy task, only a main effect of tDCS type was found (*F₁,₇₄* = 5.06, *p* = 0.027, *η_p_^2^* = 0.06), with larger amplitudes following real versus sham stimulation, regardless of target site. Collectively, these ERP findings demonstrate that rTPJ-tDCS robustly enhances neural differentiation of painful versus nonpainful stimuli, whereas lDLPFC stimulation shows no such effect, particularly in the somatic pain empathy context.

**Figure 4.**
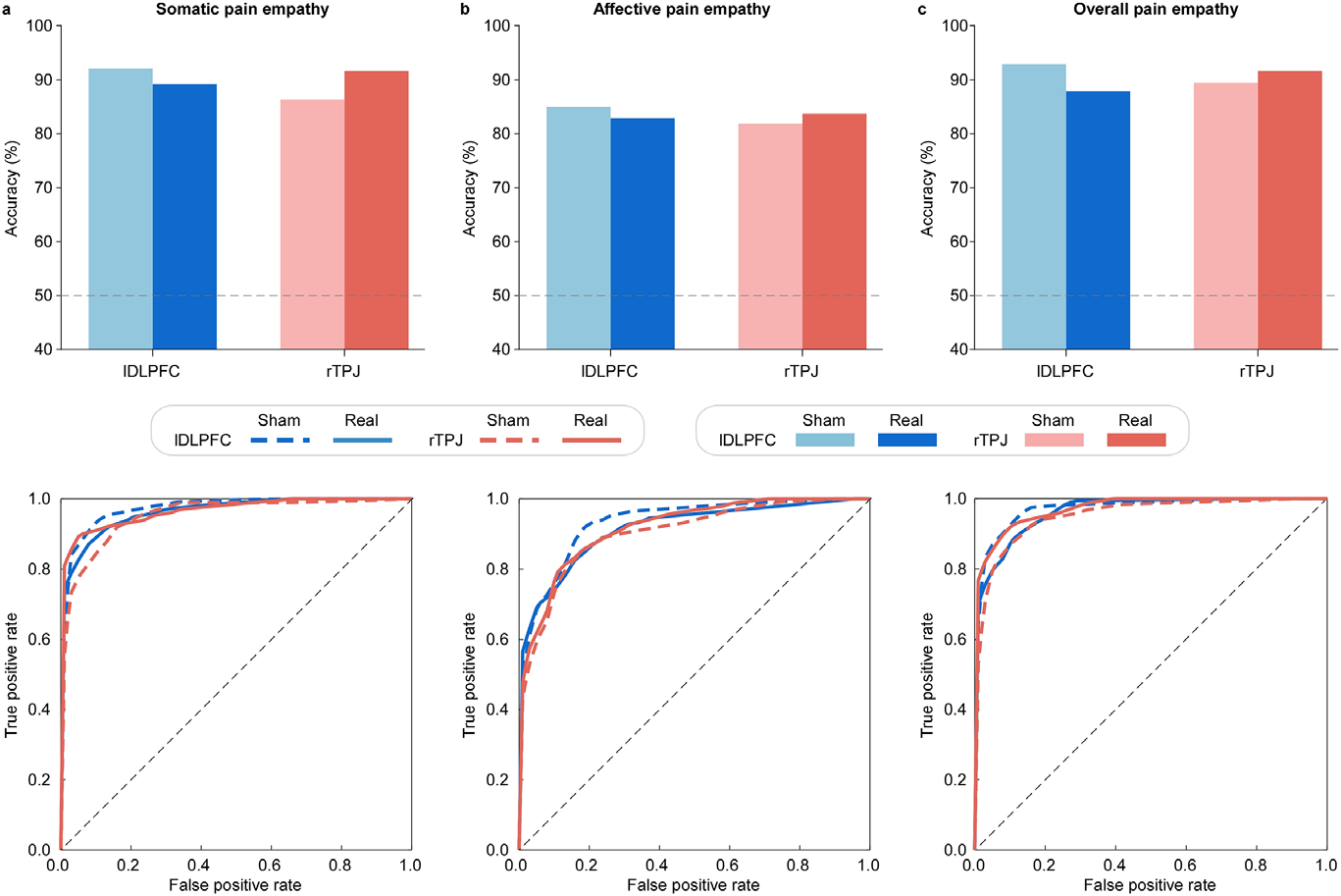
Decoding accuracy of painful versus nonpainful stimuli. Classification performance is shown for (a) somatic, (b) affective, and (c) overall pain empathy conditions. In each panel, the upper row displays classification accuracies (bar plots) for four stimulation conditions: real (darker bars) and sham (lighter bars) stimulation over the lDLPFC (blue) and rTPJ (red). The dashed horizontal line marks chance-level performance (50%). The lower row presents corresponding ROC curves, with solid lines for real stimulation and dashed lines for sham. Classifiers were trained on LPP amplitudes and behavioral ratings to distinguish painful from nonpainful stimuli. Compared with sham, rTPJ-tDCS increased decoding accuracy across all tasks, whereas lDLPFC-tDCS decreased accuracy, underscoring dissociable effects of the two stimulation sites on the computational “readability” of empathic responses.

Figure 4 summarizes decoding performance for the somatic, affective, and overall pain empathy conditions. All five classifiers (LR, KNN, RF, SVM, and NB) exhibited comparable performance and achieved classification accuracy significantly above chance. Accordingly, decoding results are reported as the average performance across all five classifiers. Detailed performance metrics for each classifier are provided in Supplementary Table S1 and Supplementary Figure S1. Across all tasks, rTPJ-tDCS improved classification accuracy relative to sham stimulation, increasing from ~86% to ~92% in the somatic condition, ~82% to ~84% in the affective condition, and ~ 89% to ~92% in the overall condition (combining somatic and affective pain empathy conditions). By contrast, lDLPFC-tDCS consistently reduced accuracy compared with sham: somatic (~92% to ~89%), affective (~85% to ~83%), and overall (~93% to ~88%). ROC analyses showed a convergent pattern, indicating enhanced separability between painful and nonpainful states following rTPJ stimulation and reduced separability following lDLPFC stimulation. Cohen’s Kappa values mirrored these effects, confirming that the observed decoding performance exceeded chance-level agreement.

### Study 2

**Figure 5.**
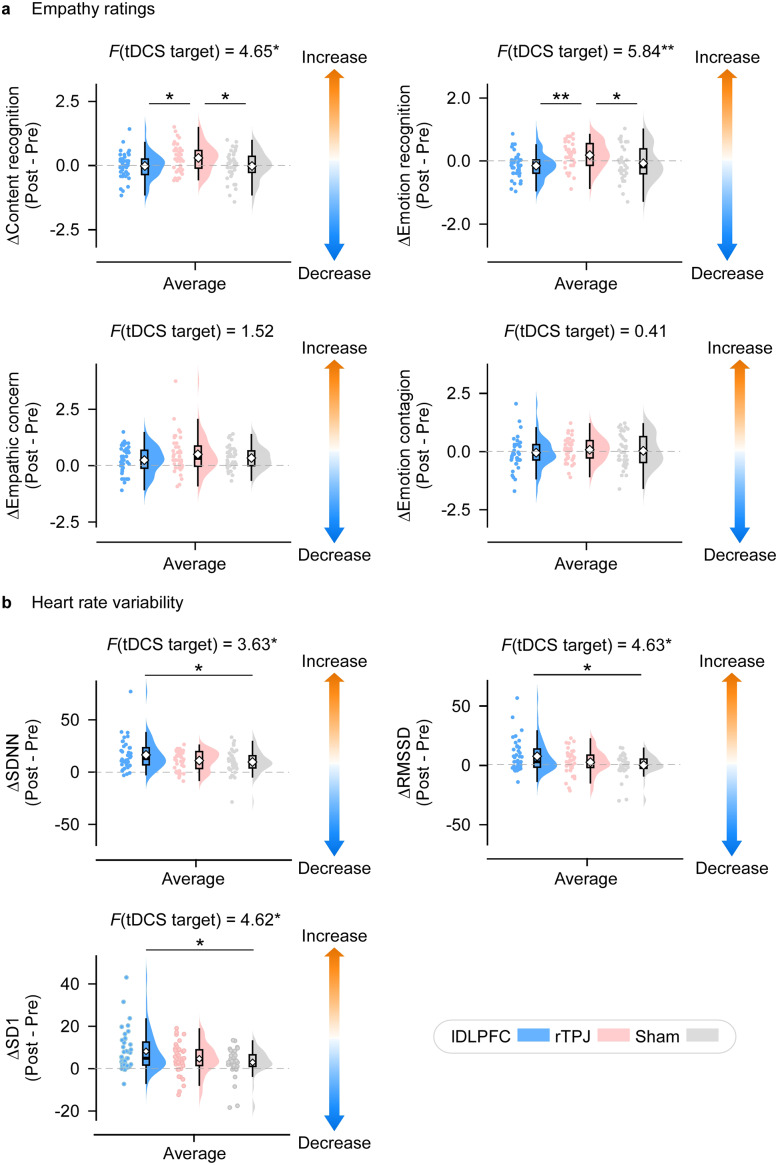
Effects of tDCS on empathy ratings and heart rate variability during the autobiographical narratives empathy task. (a) *Empathy ratings.* Changes in empathic ratings (Δ = post–pre) for autobiographical events are shown for the lDLPFC (blue), rTPJ (red), and sham (gray) groups. Raincloud plots display data distributions (violin), interquartile ranges and medians (box plots), mean values (diamonds), and individual data points. Compared with the lDLPFC-tDCS and sham stimulation, rTPJ-tDCS enhanced both content understanding and emotional recognition of the target’s autobiographical events. (b) *Heart rate variability (HRV).* Changes in HRV indices (SDNN, RMSSD, SD1; Δ = post–pre) in response to the same stimuli are shown for each group. Raincloud plots are formatted as in (a). Compared with sham stimulation, lDLPFC-tDCS significantly increased SDNN, RMSSD, and SD1, indicating enhanced parasympathetic activity. *p < 0.05; **p < 0.01.

Figure 5a shows the effects of tDCS on changes in empathic responses (Δ = post–pre) during the autobiographical narrative task. For cognitive empathy, analyses of Δcontent recognition and Δemotion recognition revealed significant main effects of stimulus type (Δcontent recognition: *F_2,117_* = 4.65, *p* = 0.011, *η_p_^2^* = 0.08; Δemotion recognition: *F_2,117_* = 5.84, *p* = 0.004, *η_p_^2^* = 0.09). Post hoc tests indicated that rTPJ-tDCS produced greater improvements in both content and emotion recognition compared with lDLPFC-tDCS (Δcontent recognition: *p* = 0.029; Δemotion recognition: *p* = 0.004) and sham stimulation (Δcontent recognition: *p* = 0.028; Δemotion recognition: *p* = 0.038).

In contrast, analyses of affective empathy indices (Δempathic concern and Δemotion contagion) revealed significant main effects of stimulus type only (Δempathic concern: *F_2,234_* = 3.94, *p* = 0.021, *η_p_^2^* = 0.03; Δemotion contagion: *F_2,234_* = 6.12, *p* = 0.003, *η_p_^2^* = 0.05). Specifically, Δempathic concern was lower for positive video clips than for neutral ones (*p* = 0.030), whereas Δemotion contagion was higher for negative stimuli than for positive stimuli (*p* = 0.002). Together, these findings indicate that rTPJ-tDCS selectively enhanced cognitive empathy, while affective responses were modulated only by stimulus valence.

Figure 5b illustrates the effects of tDCS on changes in autonomic responses (Δ = post–pre) during the autobiographical narratives empathy task. Detailed statistics of all HRV features are summarized in Supplementary Table S2. Analyses revealed significant main effects of tDCS type on of ΔSDNN (*F_2,117_* = 3.63, *p* = 0.030, *η_p_^2^* = 0.06), ΔRMSSD (*F_2,117_* = 4.63, *p* = 0.012, *η_p_^2^* = 0.07), and ΔSD1 (*F_2,117_* = 4.62, *p* = 0.012, *η_p_^2^* = 0.07). Specifically, lDLPFC-tDCS elicited greater increases in both ΔSDNN (*p* = 0.036), ΔRMSSD (*p* = 0.010) and ΔSD1 (*p* = 0.011) compared with sham stimulation, indicating enhanced parasympathetic activity and improved autonomic flexibility in response to autobiographical events. However, no significant effects were observed for the remaining HRV measures (all *p*s > 0.20).

**Figure 6.**
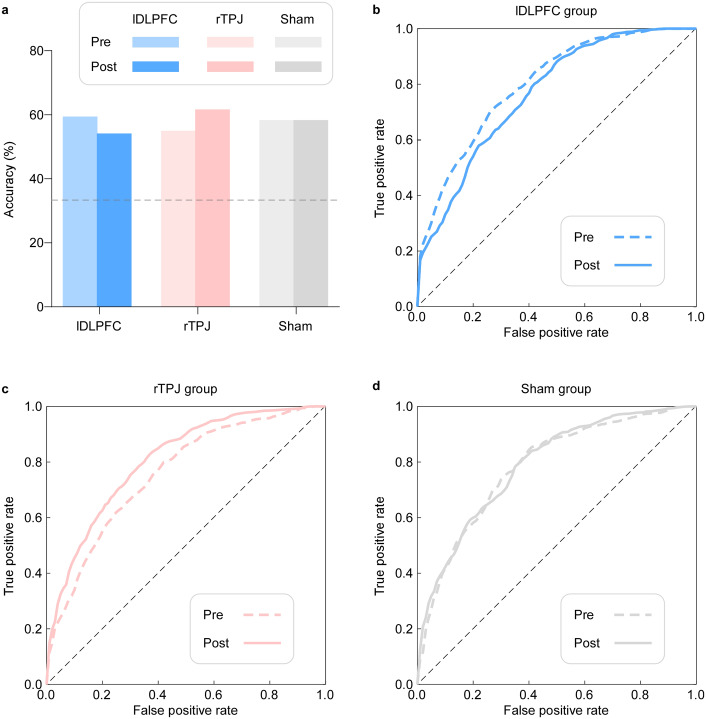
Decoding autobiographical event valence from ratings and HRV. (a) *Decoding accuracy.* Bar plots display classification accuracy for pre- (light colors) and post-stimulation (dark colors) conditions in the lDLPFC (blue), rTPJ (red), and sham (gray) groups. The dashed horizontal line indicates chance-level performance (33.3%). Relative to pre-stimulation, rTPJ-tDCS increased classification accuracy, lDLPFC-tDCS decreased accuracy, and sham stimulation showed no change. (b–d) *ROC curves.* Emotional valence (positive, neutral, and negative) was classified from behavioral and HRV features in the lDLPFC (b), rTPJ (c), and sham (d) groups. For each group, pre-stimulation (dashed lines) and post-stimulation (solid lines) curves are shown.

Figure 6 summarizes classification results across the three stimulation groups (lDLPFC, rTPJ, sham) before and after tDCS. Performance metrics for all five classifiers (LR, KNN, RF, SVM, and NB) are provided in Supplementary Table S3 and Supplementary Figure S2. In contrast to Study 1, SVM and KNN showed consistently poorer and less stable performance across conditions in this dataset, indicating reduced suitability for the feature space of Study 2. Accordingly, decoding results for Study 2 are reported as the average performance of LR, RF, and NB, which exhibited more reliable and consistent accuracy across conditions. Based on these classifiers, decoding accuracy decreased in the lDLPFC group (from ~59% pre-stimulation to ~54% post-stimulation), increased in the rTPJ group (~55% to ~62%), and remained unchanged in the sham group (~58% at both time points). ROC analyses showed a convergent pattern, with rTPJ stimulation enhancing, and lDLPFC stimulation impairing, the separability of emotional valence. Cohen’s Kappa values mirrored these effects, indicating that the observed changes exceeded chance-level agreement. Together, these results indicate a site-dependent dissociation in decoding performance: rTPJ-tDCS strengthens the discriminability of emotional valence in socially rich narrative contexts, whereas lDLPFC-tDCS reduces the discriminability.

## Discussion

The present study examined the causal roles of the rTPJ and lDLPFC in empathic processing using anodal tDCS across two complementary paradigms and multiple modalities. Three key findings emerged. First, rTPJ stimulation reliably enhanced cognitive empathy, improving discrimination in other-unpleasantness ratings and increasing empathic LPP responses in the pain empathy task, as well as strengthening self-rated content and emotion understanding in the autobiographical video task. Second, lDLPFC stimulation did not alter explicit empathic ratings but selectively increased HRV during the narrative task, consistent with a role in autonomic emotion regulation. Third, decoding analyses integrating behavioral and physiological data showed that rTPJ stimulation increased the decoding performances in predicting targets’ emotional states, whereas lDLPFC stimulation reduced decoding performance, suggesting attenuation of overt empathic signals. Together, these findings indicate dissociable, target-specific effects of neuromodulation: rTPJ stimulation supports a context-general enhancement of representing others’ states, whereas lDLPFC stimulation preferentially modulates physiological processes associated with self-oriented emotion regulation during empathic engagement.

Across two distinct paradigms, our findings highlight the rTPJ as a robust, context-general target for enhancing cognitive empathy. In the pain empathy task, rTPJ stimulation increased cognitive empathic ratings toward others’ suffering, reflected in greater discrimination of others’ unpleasantness when interpreting static images of painful versus nonpainful scenarios. At the neural level, this facilitation was indexed by amplified early and late LPP amplitudes, suggesting enhanced attentional allocation to socially salient cues and deeper evaluative processing of others’ affective states (Coll, 2018; Hajcak & Foti, 2020; Hajcak, MacNamara, & Olvet, 2010). In the autobiographical narrative task, rTPJ stimulation selectively increased self-rated content and emotion understanding, reflecting a stronger subjective sense of grasping the protagonist’s experiences and feelings. These results together indicate that rTPJ modulation benefits multiple stages of cognitive empathy, ranging from rapid perceptual differentiation to higher-order semantic and affective integration, and that these effects generalize across task types and stimulus modalities. Our findings extend prior work implicating the rTPJ as a core node of the mentalizing network (Saxe & Kanwisher, 2003; Schurz et al., 2014), critically involved in perspective-taking, self-other distinction, and social inference.

Previous neuromodulation studies targeting the rTPJ have reported heterogeneous effects on empathy- and perspective-taking–related processes, ranging from facilitation to null or inhibitory outcomes (e.g. Coll, Tremblay, & Jackson, 2017; Mai et al., 2016; Santiesteban, Banissy, Catmur, & Bird, 2012; van Elk, Duizer, Sligte, & van Schie, 2017). Such variability likely reflects differences in stimulation parameters, task demands, and outcome measures. Notably, studies reporting impaired performance often employed cathodal tDCS or low-frequency repetitive transcranial magnetic stimulation (Coll, Tremblay, & Jackson, 2017; Hartmann et al., 2024; Mai et al., 2016; Miller, Xia, & Hastings, 2020), protocols generally associated with reduced cortical excitability. By contrast, the present study applied high-definition anodal tDCS over the rTPJ, which is typically linked to increased cortical excitability and may therefore be more consistent with facilitatory effects on cognitive empathy (Nitsche & Paulus, 2000). Beyond stimulation parameters, differences in task demands and measurement strategies may further contribute to divergent findings. Many prior studies relied on paradigms probing relatively isolated components of mentalizing (e.g. imitation inhibition or momentary perspective judgments) and assessed performance primarily using reaction time or accuracy (Mai et al., 2016; Martin et al., 2020; Miller, Xia, & Hastings, 2020; Nobusako et al., 2017; Santiesteban, Banissy, Catmur, & Bird, 2012). In contrast, the present study combined pain-related paradigms with an autobiographical narrative task requiring sustained self-other distinction, semantic integration, and inference of others’ internal states over extended time scales, and employed a multimodal assessment framework integrating behavioral, EEG, and autonomic measures. Together, these features may place greater demands on rTPJ-mediated integrative processes and increase sensitivity to certain facilitatory neuromodulation effects.

In contrast to the context-general effects observed for rTPJ stimulation, lDLPFC-tDCS exerted a more selective influence, primarily reflected in enhanced HRV during the autobiographical narrative task. This physiological profile is consistent with the established role of the lDLPFC in top-down emotion regulation, executive control, and modulation of self-oriented distress (Dorfel et al., 2014; Zhao et al., 2021). Nevertheless, these physiological changes were not accompanied by reliable alterations in explicit empathy ratings. This dissociation suggests that lDLPFC stimulation does not directly enhance empathic inference or emotional sharing, but rather preferentially modulates internal regulatory states engaged during emotionally salient social contexts. Accordingly, lDLPFC stimulation appears to enhance regulatory capacity, rather than altering the experiential or representational components of empathy. This interpretation aligns with converging evidence indicating that the DLPFC plays a specialized role in emotion regulation and cognitive control (Golkar et al., 2012; Morawetz et al., 2016; Ochsner & Gross, 2005), rather than in the direct representation of others’ mental or emotional states (Fan, Duncan, de Greck, & Northoff, 2011; Schurz et al., 2021). Framed in this way, the present findings also help reconcile prior reports of inconsistent or null effects of DLPFC-tDCS on subjective empathy measures (Rego et al., 2015; Snowdon & Cathcart, 2018; Wang, Wang, Hu, & Li, 2014), suggesting that variability across studies may reflect the region’s relatively circumscribed contribution to automatic regulatory processes rather than to overt empathic experience.

A key contribution of this study is the use of decoding analyses as a computational complement to conventional behavioral and physiological measures. Decoding performance indexes empathic processing by quantifying how reliably an observer’s integrated behavioral and physiological responses predict the target’s emotional state. Whereas traditional analyses evaluate individual measures in isolation, decoding captures cross-modal coherence, thereby indexing the alignment between internal empathic states and external emotional cues. Higher decoding accuracy thus reflects stronger coupling and clearer external expression of empathic responses, whereas lower accuracy indicates weakened alignment or attenuated expressivity (Kragel, Koban, Barrett, & Wager, 2018; Woo, Chang, Lindquist, & Wager, 2017). This approach corroborated the univariate findings by revealing convergent modulation patterns at the multivariate level. Specifically, rTPJ stimulation not only enhanced explicit empathy measures but also increased decoding accuracy, suggesting strengthened coherence and external interpretability of empathic responses. In contrast, lDLPFC stimulation increased HRV without altering subjective ratings, yet was associated with reduced decoding accuracy, indicating that enhanced regulatory flexibility may attenuate overt emotional signals and render empathic states less externally decodable. Together, the decoding results show that neuromodulation reshapes empathy by reorganizing the alignment between observers’ behavioral/physiological responses and targets’ emotional states.

The present findings advance empathy theory by providing causal support for a dual-route framework in which partially dissociable neural circuits contribute to other-oriented and self-oriented components of empathic processing (Davis, 1983; Decety & Lamm, 2006; Zaki & Ochsner, 2012). Although prior neuroimaging studies have linked the rTPJ to perspective-taking and mental state attribution (Decety & Lamm, 2007; Saxe & Kanwisher, 2003), and the lDLPFC to top-down emotion regulation and the control of self-oriented distress (Dorfel et al., 2014; Morawetz, Bode, Derntl, & Heekeren, 2017; Zilverstand, Parvaz, & Goldstein, 2017), this evidence has been largely correlational. By directly modulating these regions, the present tDCS results provide causal evidence consistent with this functional dissociation. Specifically, anodal rTPJ tDCS was associated with enhanced indices of cognitive empathy across both pain-related and narrative-based contexts, supporting a context-general role in representing others’ emotional states. In contrast, lDLPFC tDCS selectively increased autonomic flexibility, as indexed by HRV, without reliable changes in explicit empathy ratings, pointing to a more circumscribed contribution to regulatory processes engaged during empathic contexts. These findings indicate that the rTPJ and lDLPFC support empathy through complementary yet dissociable mechanisms, moving beyond correlational associations to establish their causal roles in empathic processing.

Beyond theoretical refinement, our findings also carry clear translational significance. By demonstrating dissociable, target-specific effects of rTPJ and lDLPFC stimulation on distinct components of empathy, our results suggest that empathy-related difficulties may arise from different underlying mechanisms and therefore require differentiated neuromodulation strategies. Specifically, rTPJ stimulation selectively enhanced other-oriented, representational aspects of empathy across contexts, highlighting its potential relevance for conditions characterized by impaired perspective-taking and mental state attribution, such as autism spectrum disorder (Mazza et al., 2014) or schizophrenia (Bora, Yucel, & Pantelis, 2009). In contrast, lDLPFC stimulation primarily modulated autonomic regulation, suggesting potential utility for populations in which excessive personal distress or emotion dysregulation constrains adaptive empathic engagement, such as caregivers or individuals with post-traumatic stress disorder (Couette et al., 2020; Mazza et al., 2015).

Several limitations should be noted. First, all experiments were conducted in healthy participants, which limits direct generalization to clinical populations with empathy-related difficulties. Although this design enabled the identification of causal, target-specific effects under controlled conditions, future studies are needed to test whether similar modulation profiles extend to clinical groups. Second, stimulation effects were assessed following single-session tDCS, leaving open the question of whether repeated stimulation induces more durable changes in empathic processing. Finally, although the sample size is comparable to prior tDCS studies on empathy (La Malva et al., 2024; Li et al., 2021; Mai et al., 2016; Martin et al., 2020), larger samples will be important for more systematically characterizing individual differences in stimulation responsiveness and for guiding the development of personalized neuromodulation protocols.

## Conclusion

This study examined how anodal tDCS over the rTPJ and lDLPFC modulates empathic processing. rTPJ stimulation was associated with enhanced cognitive empathy across both picture- and narrative-based tasks, as reflected by higher empathy-related ratings, increased LPP amplitudes, and improved decoding of others’ emotional states. In contrast, lDLPFC stimulation selectively increased HRV during the narrative-based task, indicating enhanced autonomic regulatory capacity, and was accompanied by reduced decodability of empathic signals. By integrating behavioral, neurophysiological, and computational measures, these findings provide neuromodulation evidence consistent with dissociable contributions of the rTPJ and lDLPFC to empathy, with the rTPJ more closely linked to representational aspects of understanding others’ states and the lDLPFC more closely linked to regulatory processes. Together, these results refine mechanistic models of empathy and inform the development of target-specific neuromodulation strategies for empathy-related deficits and regulation needs.

## Supporting information

10.1017/S0033291726104218.sm001 Li et al. supplementary material Li et al. supplementary material

## Supplementary material

The supplementary material for this article can be found at http://doi.org/10.1017/S0033291726104218.
