# Supplementary material for: Modulating empathy with tDCS: dissociable roles of rTPJ and lDLPFC
Source: Psychol Med. 2026 Apr 28;56:e121. doi: 10.1017/S0033291726104218 (PMC13125934; doi:10.1017/S0033291726104218)
Supplement: Li et al. supplementary material [file S0033291726104218sup001.docx]

**Supplementary Materials**

**Table S1. Classification performance across five machine learning classifiers for somatic, affective, and overall pain empathy conditions in Study 12**

**Figure S1. Decoding accuracy of painful versus nonpainful stimuli across five machine learning classifiers in Study 13**

**Table S2. Changes heart rate variability (HRV) indices during the autobiographical narrative empathy task in Study 24**

**Table S3. Classification performance across five machine learning classifiers for the three stimulation groups in Study 25**

**Figure S2. Decoding autobiographical event valence from behavioral ratings and HRV features across five different classifiers in Study 26**

**Table S1.** **Classification performance across five machine learning classifiers for somatic, affective, and overall pain empathy conditions in Study 1**

| **Target** | **Type** | **Model** | **Somatic pain empathy** | | | **Affective pain empathy** | | | **Overall pain empathy** | | |
| --- | --- | --- | --- | --- | --- | --- | --- | --- | --- | --- | --- |
|  |  |  | **Accuracy (%)** | **Kappa** | **AUC** | **Accuracy (%)** | **Kappa** | **AUC** | **Accuracy (%)** | **Kappa** | **AUC** |
| lDLPFC | Real | RF | 90.79 | 0.82 | 0.98 | 86.84 | 0.74 | 0.95 | 93.42 | 0.87 | 0.98 |
|  |  | SVM | 88.16 | 0.76 | 0.96 | 82.89 | 0.66 | 0.92 | 86.84 | 0.74 | 0.96 |
|  |  | LR | 92.11 | 0.84 | 0.98 | 86.84 | 0.74 | 0.96 | 88.16 | 0.76 | 0.97 |
|  |  | KNN | 85.53 | 0.71 | 0.91 | 72.37 | 0.45 | 0.76 | 80.26 | 0.61 | 0.91 |
|  |  | NB | 89.47 | 0.79 | 0.98 | 85.53 | 0.71 | 0.95 | 90.79 | 0.82 | 0.99 |
|  | Sham | RF | 96.05 | 0.92 | 0.99 | 86.84 | 0.74 | 0.97 | 96.05 | 0.92 | 0.99 |
|  |  | SVM | 92.11 | 0.84 | 0.97 | 81.58 | 0.63 | 0.93 | 89.47 | 0.79 | 0.98 |
|  |  | LR | 94.74 | 0.89 | 0.99 | 88.16 | 0.76 | 0.96 | 96.05 | 0.92 | 0.99 |
|  |  | KNN | 84.21 | 0.68 | 0.92 | 81.58 | 0.63 | 0.83 | 86.84 | 0.74 | 0.91 |
|  |  | NB | 93.42 | 0.87 | 0.97 | 86.84 | 0.74 | 0.95 | 96.05 | 0.92 | 0.98 |
| rTPJ | Real | RF | 96.05 | 0.92 | 0.99 | 85.53 | 0.71 | 0.93 | 96.05 | 0.92 | 0.99 |
|  |  | SVM | 90.79 | 0.82 | 0.96 | 86.84 | 0.74 | 0.93 | 93.42 | 0.87 | 0.98 |
|  |  | LR | 94.74 | 0.89 | 1.00 | 85.53 | 0.71 | 0.94 | 92.11 | 0.84 | 0.98 |
|  |  | KNN | 82.89 | 0.66 | 0.89 | 73.68 | 0.47 | 0.82 | 82.89 | 0.66 | 0.92 |
|  |  | NB | 93.42 | 0.87 | 0.99 | 86.84 | 0.74 | 0.95 | 93.42 | 0.87 | 0.99 |
|  | Sham | RF | 92.11 | 0.84 | 0.98 | 84.21 | 0.68 | 0.92 | 97.37 | 0.95 | 0.98 |
|  |  | SVM | 85.53 | 0.71 | 0.95 | 80.26 | 0.61 | 0.90 | 86.84 | 0.74 | 0.97 |
|  |  | LR | 88.16 | 0.76 | 0.98 | 85.53 | 0.71 | 0.96 | 89.47 | 0.79 | 0.98 |
|  |  | KNN | 75.00 | 0.50 | 0.86 | 72.37 | 0.45 | 0.76 | 77.63 | 0.55 | 0.84 |
|  |  | NB | 90.79 | 0.82 | 0.97 | 86.84 | 0.74 | 0.92 | 96.05 | 0.92 | 0.98 |

Notes: RF, Random Forest; SVM, Support Vector Machine; LR, Logistic Regression; KNN, k-Nearest Neighbors; NB, Naïve Bayes.


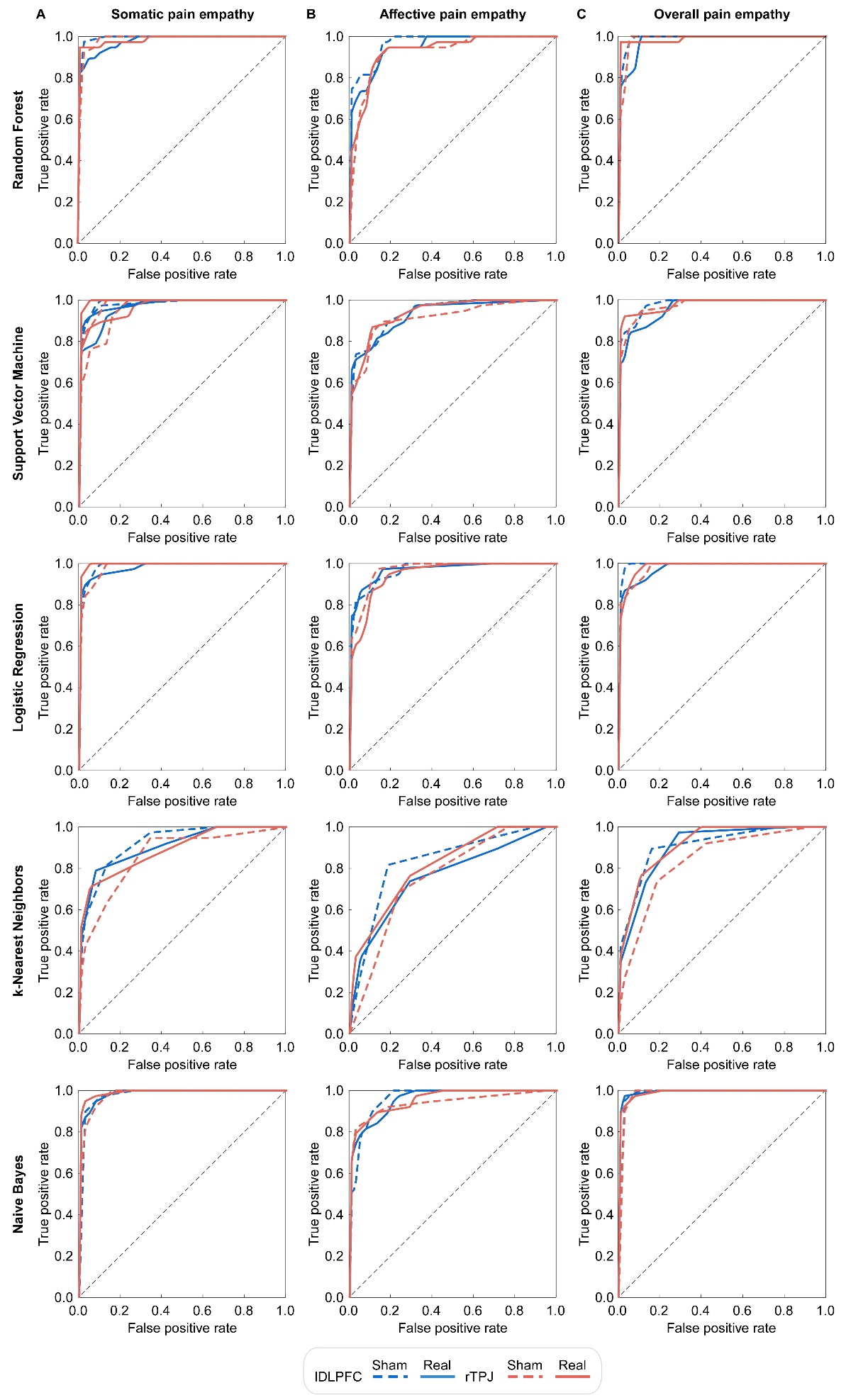


**Figure S1. Decoding accuracy of painful versus nonpainful stimuli across five machine learning classifiers in Study 1**

**Table S2. Changes heart rate variability (HRV) indices during the autobiographical narrative empathy task in Study 2**

| HRV feature | lDLPFC group | rTPJ group | Sham group | Statistics |
| --- | --- | --- | --- | --- |
| ΔHR | –4.79 ± 0.77 | –4.21 ± 0.70 | –4.51 ± 0.64 | *F_2,117_* = 0.17, *p* = 0.848, *η_p_²* = 0.003 |
| ΔSDNN | 17.72 ± 2.31 | 12.32 ± 1.47 | 10.95 ± 1.76 | *F_2,117_* = 3.63, *p* = 0.030, *η_p_²* = 0.06 |
| ΔRMSSD | 11.46 ± 2.15 | 6.46 ± 1.59 | 4.06 ± 1.45 | *F_2,117_* = 4.63, *p* = 0.012, *η_p_²* = 0.07 |
| ΔpNN50 | 7.36 ± 1.56 | 5.01 ± 1.36 | 5.01 ± 1.23 | *F_2,117_* = 0.93, *p* = 0.397, *η_p_²* = 0.02 |
| ΔLF | 591.06 ± 110.03 | 464.34 ± 63.26 | 553.56 ± 97.51 | *F_2,117_* = 0.50, *p* = 0.610, *η_p_²* = 0.01 |
| ΔHF | 284.52 ± 129.50 | 132.35 ± 87.55 | 129.71 ± 40.44 | *F_2,117_* = 0.90, *p* = 0.408, *η_p_²* = 0.02 |
| ΔLF/HF ratio | 0.32 ± 0.17 | –0.01 ± 0.22 | 0.64 ± 0.20 | *F_2,117_* = 2.66, *p* = 0.074, *η_p_²* = 0.04 |
| ΔSample entropy | –0.13 ± 0.02 | –0.08 ± 0.02 | –0.12 ± 0.03 | *F_2,117_* = 1.39, *p* = 0.253, *η_p_²* = 0.02 |
| ΔSD1 | 8.14 ± 1.52 | 4.59 ± 1.13 | 2.89 ± 1.03 | *F_2,117_* = 4.62, *p* = 0.012, *η_p_²* = 0.07 |
| ΔSD2 | 23.58 ± 3.09 | 17.19 ± 1.90 | 15.65 ± 2.38 | *F_2,117_* = 2.82, *p* = 0.064, *η_p_²* = 0.05 |
| ΔSD1/SD2 ratio | –0.03 ± 0.02 | –0.04 ± 0.02 | –0.05 ± 0.02 | *F_2,117_* = 0.68, *p* = 0.510, *η_p_²* = 0.01 |

Notes: Data are expressed using Mean ± SEM. Statistics were performed using the one-way analysis of variance. Δ = post – pre.

**Table S3. Classification performance across five machine learning classifiers for the three stimulation groups in Study 2**

| **Target** | **Time** | **Model** | **Accuracy (%)** | **Kappa** | **AUC** |
| --- | --- | --- | --- | --- | --- |
| lDLPFC | Pre | RF | 61.67 | 0.43 | 0.79 |
|  |  | SVM | 33.33 | 0.00 | 0.50 |
|  |  | LR | 57.50 | 0.36 | 0.80 |
|  |  | KNN | 39.17 | 0.09 | 0.54 |
|  |  | NB | 59.17 | 0.39 | 0.79 |
|  | Post | RF | 52.50 | 0.29 | 0.76 |
|  |  | SVM | 30.83 | -0.04 | 0.51 |
|  |  | LR | 57.50 | 0.36 | 0.77 |
|  |  | KNN | 33.33 | 0.00 | 0.55 |
|  |  | NB | 52.50 | 0.29 | 0.75 |
| rTPJ | Pre | RF | 59.17 | 0.39 | 0.78 |
|  |  | SVM | 34.17 | 0.01 | 0.48 |
|  |  | LR | 51.67 | 0.28 | 0.74 |
|  |  | KNN | 30.83 | -0.04 | 0.49 |
|  |  | NB | 54.17 | 0.31 | 0.75 |
|  | Post | RF | 65.00 | 0.48 | 0.82 |
|  |  | SVM | 25.00 | -0.13 | 0.55 |
|  |  | LR | 57.50 | 0.36 | 0.79 |
|  |  | KNN | 26.67 | -0.10 | 0.46 |
|  |  | NB | 62.50 | 0.44 | 0.80 |
| Sham | Pre | RF | 59.17 | 0.39 | 0.77 |
|  |  | SVM | 31.67 | -0.02 | 0.50 |
|  |  | LR | 61.67 | 0.43 | 0.81 |
|  |  | KNN | 33.33 | 0.00 | 0.49 |
|  |  | NB | 54.17 | 0.31 | 0.75 |
|  | Post | RF | 56.67 | 0.35 | 0.78 |
|  |  | SVM | 35.83 | 0.04 | 0.50 |
|  |  | LR | 65.00 | 0.48 | 0.83 |
|  |  | KNN | 28.33 | -0.07 | 0.49 |
|  |  | NB | 53.33 | 0.30 | 0.75 |

Notes: RF, Random Forest; SVM, Support Vector Machine; LR, Logistic Regression; KNN, k-Nearest Neighbors; NB, Naïve Bayes.


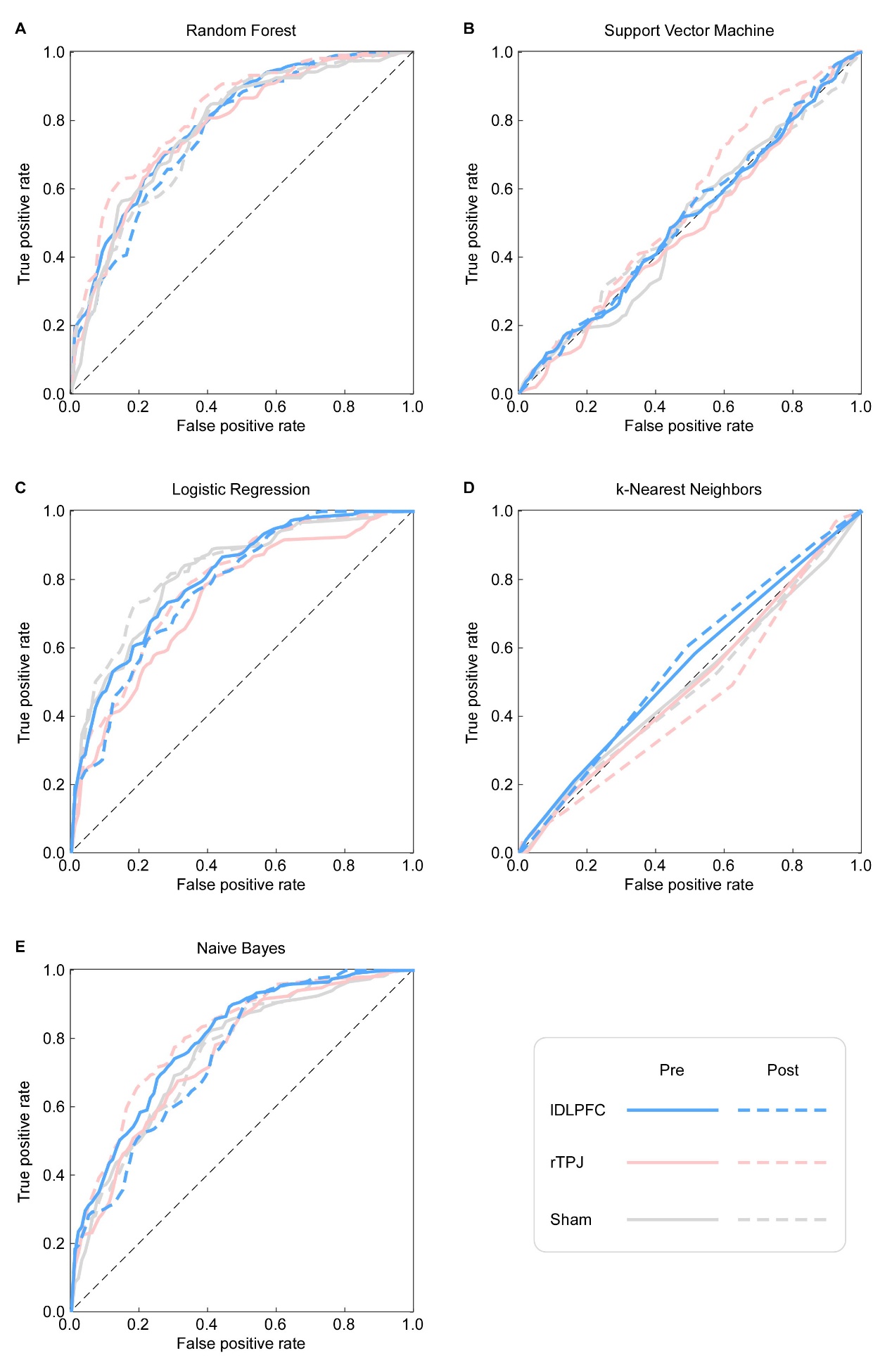


**Figure S2. Decoding autobiographical event valence from behavioral ratings and HRV features across five different classifiers in Study 2**
